# Production and characterization of lipopeptide biosurfactant from a new strain of *Pseudomonas antarctica* 28E using crude glycerol as a carbon source

**DOI:** 10.1039/d3ra03408a

**Published:** 2023-08-11

**Authors:** Dominika Ciurko, Alif Chebbi, Mateusz Kruszelnicki, Hanna Czapor-Irzabek, Aneta K. Urbanek, Izabela Polowczyk, Andrea Franzetti, Tomasz Janek

**Affiliations:** a Department of Biotechnology and Food Microbiology, Wrocław University of Environmental and Life Sciences 51-630 Wrocław Poland tomasz.janek@upwr.edu.pl +48-71-320-7734; b Department of Science, Roma Tre University 00146 Rome Italy; c Department of Process Engineering and Technology of Polymers and Carbon Materials, Wroclaw University of Science and Technology 50-370 Wrocław Poland; d Laboratory of Elemental Analysis and Structural Research, Wroclaw Medical University 50-556 Wroclaw Poland; e Faculty of Biotechnology, University of Wroclaw 50-383 Wroclaw Poland; f Department of Earth and Environmental Sciences – DISAT, University of Milano-Bicocca 20126 Milano Italy

## Abstract

*Pseudomonas* is a cosmopolitan genus of bacteria found in soil, water, organic matter, plants and animals and known for the production of glycolipid and lipopeptide biosurfactants. In this study bacteria (laboratory collection number 28E) isolated from soil collected in Spitsbergen were used for biosurfactant production. 16S rRNA sequencing and matrix-assisted laser desorption/ionization time-of-flight mass spectrometry (MALDI-TOF) revealed that this isolate belongs to the species *Pseudomonas antarctica*. In the present study, crude glycerol, a raw material obtained from several industrial processes, was evaluated as a potential low-cost carbon source to reduce the costs of lipopeptide production. Among several tested glycerols, a waste product of stearin production, rich in nitrogen, iron and calcium, ensured optimal conditions for bacterial growth. Biosurfactant production was evidenced by a reduction of surface tension (ST) and an increase in the emulsification index (*E*_24_%). According to Fourier-transform infrared spectroscopy (FTIR) and electrospray ionization mass spectrometry (ESI-MS), the biosurfactant was identified as viscosin. The critical micelle concentration (CMC) of lipopeptide was determined to be 20 mg L^−1^. Interestingly, viscosin production has been reported previously for *Pseudomonas viscosa*, *Pseudomonas fluorescens* and *Pseudomonas libanensis*. To the best of our knowledge, this is the first report on viscosin production by a *P. antarctica* 28E. The results indicated the potential of crude glycerol as a low-cost substrate to produce a lipopeptide biosurfactant with promising tensioactive and emulsifying properties.

## Abstract 2

Stearin-derived waste glycerol as an economic substrate for biosurfactant production using *Pseudomonas antarctica* isolated from the Arctic Archipelago of Svalbard.

## Graphical abstract

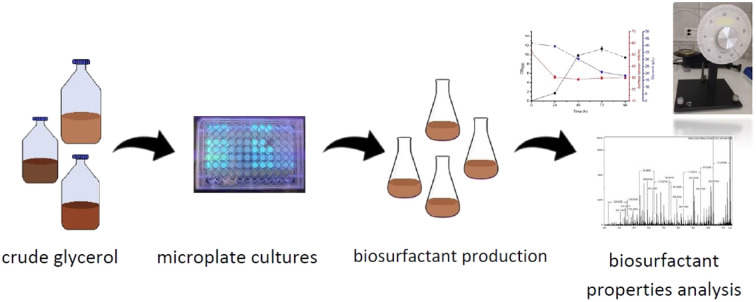

## Introduction

Surfactants are described as active ingredients, found in household and industrial-scale cleaning agents.^1^ Their activity derives from the surface or interfacial tension reduction properties, which increase the solubility of non-aqueous phase liquids. Despite their numerous benefits, the high toxicity of surfactants is not without significance.^2^ The negative environmental impact is related to the production process, occurring *via* chemical synthesis. Moreover, surfactants' recalcitrant and persistent nature entails a slow rate of degradation. Despite high environmental toxicity, chemically derived surfactants are still in wide scale application.^3^ However, in a time of ecological crisis there is a growing demand for environmentally friendly agents.^1^ Therefore, biosurfactants are increasingly taking the place of their synthetic counterparts. Biosurfactants are surface-active compounds physiologically secreted by microorganisms for utilization of hydrocarbons.^4^ Microbial surfactants exhibit superior properties compared to chemically manufactured alternatives, of which the most important is outstanding surface activity, stability in a wide range of pH and temperature, biodegradability, low toxicity and extraordinary emulsifying and demulsifying activity.^5^

Numerous genera have been described for biosurfactant production, with *Bacillus* spp., *Acinetobacter* spp., *Rhodococcus* spp., *Halomonas* spp. and *Candida* spp. being the best-known biosurfactant producers, as well as *Pseudomonas*, described as a dominant genus involved in biosurfactant synthesis.^6^*Pseudomonas* is a cosmopolitan microorganism found in miscellaneous habitats including soil, water, organic matter, plants and animals.^7^ Due to its metabolic complexity, *Pseudomonas* is capable of synthesizing both glycolipid and lipopeptide biosurfactants.^8,9^ Known for its higher rhamnolipid production, *P. fluorescens* is at the same time extensively studied for lipopeptide synthesis.^10^ Lipopeptide biosurfactants of *Pseudomonas* spp. are known to exhibit great diversity.^11^ Their differences are mainly manifested in the length and composition of the fatty acid tail as well as in the number, type, and configuration of the amino acids in the peptide chain.^12^ The complex group of *Pseudomonas* lipopeptides, originally represented by viscosin, amphisin, tolaasin and syringomycin, has been recently expanded by several newly identified compounds.^13^ Therefore, arthrofactin of *Pseudomonas* sp. strain MIS38, putisolvins produced by *Pseudomonas putida*, orfamide of *P. fluorescens* strain Pf-5 and pseudodesmins of an unidentified *Pseudomonas* isolate have recently become the objects of intense research.^14^ In fact, the extensively studied group of viscosins has so far included 17 characterized compounds, represented by viscosin, viscosinamide, WLIP (white line-inducing principle) pseudodesmin, massetolide and pseudophomins.^15^ These lipopeptides are composed of a peptide chain of nine amino acids, typically attached to 3-hydroxydecanoic acid (3-HDA).^15^ The structure is cyclized by the ester bound, which connects the C-terminus of the peptide chain with the alcohol function of the third threonine side chain. Viscosin-like lipopeptides are varied in terms of the hydrophobic amino acid located at positions 4 and 9, where Leu, Ile or Val may be present. In addition, the fatty acid tail may include from 10 to 12 carbon atoms.^15,16^ To date, viscosin production has been demonstrated for a limited number of *Pseudomonas* species. The first report of Groupé *et al.*^17^ described the isolation of the antibiotic compound, and its activity against pathogenic and saprophytic mycobacteria. The antibiotic was secreted in *P. viscosa* culture and therefore named viscosin. Later, Neu *et al.*^18^ determined the surface active properties of viscosin. Lipopeptide purified from *P. fluorescens* culture showed the ability to reduce ST to the value of 26.5 mN m^−1^. The critical micelle concentration (CMC) was determined to be 0.15 mg ml^−1^. Further research of Hildebrand *et al.*^19^ evidenced viscosin production in the culture of *P. fluorescens* (P/SH10-3B-A7B). Once again, the significant surface-active properties of viscosin were confirmed. Further, De Bruijn *et al.*^20^ documented the significant role of viscosin-like lipopeptide, extracted from *P. fluorescens* SBW25 culture medium, in motility and biofilm formation. Finally, efficient production of viscosin lipopeptide was documented for *Pseudomonas libanensis* M9-3, isolated as a result of a hydroponic greenhouse study.^21^

Given the dearth of knowledge regarding the lipopeptides' production by the *Pseudomonas* genus, this paper aims at describing, for the first time, the capability of a *P. antarctica* 28E strain to produce the lipopeptide biosurfactant viscosin. The purpose of this study was to evaluate the conversion of crude glycerol to viscosin using *P. antarctica* 28E. Additionally, the molecular structure and physicochemical properties (including CMC) and the wettability alteration (contact angle determination) of the produced lipopeptide were studied.

## Materials and methods

### Identification studies

*P. antarctica* strain 28E, previously isolated from soil samples collected along western and central parts of Spitsbergen, Svalbard Archipelago^22^ was identified by the analysis of the 16S rRNA gene. To achieve better strain identification, matrix-assisted laser desorption/ionization time-of-flight mass spectrometry (MALDI-TOF) was also employed. For sequencing, genomic bacterial DNA was isolated using the Bacterial & Yeast Genomic DNA Purification Kit (EURx, Poland). The concentration of the isolated DNA sample was determined using Nanodrop (WPA Biowave II, USA). PCR of isolated DNA was performed in a total value of 20 μL of reaction mixture, containing 10 μL of PCR MasterMix 2X (Thermo Scientific, USA), 1 μL of 27F (5′-AGAGTTTGATCCTGGCTCAG-3′) and 1492R (5′-GGTTACCTTGTTACGACTT3′) primers, genomic DNA, applied in a concentration of 50–100 ng, and nuclease-free water. PCR MasterMix 2X contained Taq DNA polymerase (0.05 U μL^−1^), reaction buffer, MgCl_2_ (4 mM), and 0.4 mM of each dNTP. Reaction of polymerase was initiated by a pre-denaturation step performed at 95 °C for 5 min, followed by 30 cycles of denaturation (95 °C, 20 s), annealing (55 °C, 30 s) and elongation (72 °C, 2 min). The reaction was terminated with an extended elongation (72 °C, 5 min). The PCR products were subjected to agarose gel electrophoresis, purified using a Gel-Out Kit for DNA extraction (A&A Biotechnology, Poland), and their nucleotide sequence was determined by the Genomed S.A. sequencing service (Warsaw, Poland).

Identification of *P. antarctica* 28E by the MALDI TOF MS method was carried out in the Microbiological Laboratory in the Jagiellonian Center of Innovation in Cracow, using a MALDI-TOF Microflex LT mass spectrometer by Bruker (Bruker, USA). Sequences of 16S rRNA of *P. antarctica* 28E and results of MALDI-TOF study were then analyzed with the Basic Local Alignment Search Tool (BLAST) program, available on the National Center for Biotechnology Information (NCBI) website.

### Media and growth conditions

The bacterial strain *P. antarctica* 28E was stored at the Department of Biotechnology and Food Microbiology, Wrocław University of Environmental and Life Sciences (Wrocław, Poland) in the form of glycerol stock (20% v/v) at −80 °C and, when needed, cultured in Luria–Bertani broth (LB; 10 g L^−1^ of tryptone, 5 g L^−1^ of yeast extract, and 10 g L^−1^ of NaCl; A&A Biotechnology, Poland) for 20 h with orbital agitation (180 rpm) at 28 °C.

Initially, *P. antarctica* 28E was cultured in the mineral salt medium (MSM) supplemented with crude glycerols, to determine the optimal substrate for bacterial growth. Glycerols were applied at two concentrations, 2% (w/v) and 4% (w/v). MSM composed of (g L^−1^) NaNO_3_ (2), Na_2_HPO_4_ (0.9), MgSO_4_ × 7H_2_O (0.4), KH_2_PO_4_ (0.7), CaCl_2_ × 2H_2_O (0.1), FeSO_4_ × 7H_2_O (0.001) was adjusted to pH 7.4–7.6 using 5 M NaOH and sterilized. Crude glycerols used in this study were generated as a result of soap production process (glycerol Racibórz (G1) (Polish oil concern PKN ORLEN)), biodiesel production (glycerol Lotos (G2) (Grupa Lotos S.A., Poland), Trzebinia (G3) and Czechowice (G4) (Grupa Azoty S.A., Poland)) and stearin production (glycerol Chorzów (G5) (Grupa Lotos S.A., Poland)), described in detail in the work of Dobrowolski *et al.*^23^ and are listed in Table 1.

**Table 1:** Composition of crude glycerol from different sources^23^

Symbol	G1	G2	G3	G4	G5
Waste product derived from	Soap production	Biodiesel production	Biodiesel production	Biodiesel production	Stearin production
Glycerol (%)	80	50	87	80	42
Nitrogen (%)	0.041	0.078	0.023	0.014	0.136
NaCl (%)	7.59	3.04	0.20	5.47	1.23
Ash (%)	8.76	3.62	0.93	6.34	1.35
Water (%)	3.6	43.42	11.85	8.16	55.3
Na (g kg^−1^)	31.63 ± 0.52	13.10 ± 0.52	0.44 ± 0.01	23.12 ± 0.76	5.22 ± 0.26
K (mg kg^−1^)	231.32 ± 4.77	72.74 ± 8.98	4388.16 ± 174.37	65.89 ± 4.88	63.137 ± 6.31
Mg (mg kg^−1^)	5.454 ± 0.44	15.26 ± 0.45	1.68 ± 0.17	22.55 ± 1.02	5.305 ± 1.06
Ca (mg kg^−1^)	52.36 ± 10.93	97.06 ± 9.44	40.27 ± 6.65	132.21 ± 5.45	461.95 ± 46.19
Fe (mg kg^−1^)	26.42 ± 1.20	2.31 ± 0.68	1.99 ± 0.17	5.91 ± 0.20	458.38 ± 45.84
Zn (mg kg^−1^)	1.30 ± 0.40	1.13 ± 0.34	0.09 ± 0.02	1.41 ± 0.13	1.251 ± 0.25
Cu (mg kg^−1^)	0.66 ± 0.08	0.12 ± 0.08	0.096 ± 0.04	0.39 ± 0.04	0.03 ± 0.01
Cl (mg kg^−1^)	46 000 ± 21.20	18 500 ± 30.00	1200 ± 10.90	33 200 ± 425.56	7400 ± 59.26

In addition, another glycerol-derived waste, previously characterized by Signori *et al.*,^24^ originating from industrial biodiesel production from palm oil (Milan (G7)) was included in this research. In addition, to estimate the suitability of crude glycerol as a potential substrate for biosurfactant synthesis, a process was additionally conducted using pure glycerol (G6) (99.5%) (Centro-Chem, Poland) as a control. The purity of crude glycerols was estimated using high-performance liquid chromatography (HPLC, UltiMate 3000, Dionex-Thermo Fisher Scientific, London, UK) equipped with a HyperRez Carbohydrate H^+^ Column (Thermo Fisher Scientific, London, UK) and a refractive index (RI) detector (Shodex, Ogimachi, Japan). Trifluoroacetic acetic acid at the concentration 25 mM was applied as an elution agent. Elution was carried out with a flow rate of 0.6 mL min^−1^ at 65 °C.^25^

### Microplate cultures

Glycerols were sterilized and added in a proper ratio to the sterile MSM. Microplate cultures were conducted in The Spark multimode microplate reader (Tecan Group Ltd) for 120 h at 28 °C with continuous orbital shaking (180 rpm). Cultures were carried out in a 96-well plate. Each well was filled with 200 μL of the medium, inoculated with 1% LB bacterial culture, performed as described in section media and growth conditions. Optical density (OD_600_) was measured each hour in 120 cycles. All measurements were carried out in triplicate.

### Studies on biosurfactant production

According to the results of microplate cultures, further investigations were performed using G5 glycerol, ensuring the best growth of *P. antarctica* 28E. The biosurfactant production was carried out in 250 mL baffled Erlenmeyer flasks containing 50 mL of MSM medium, supplemented with 2% and 4% waste glycerol. Cultures were inoculated with 1% 20 h LB bacterial inoculum and incubated at 28 °C for 96 h with orbital agitation (180 rpm). As a control, uninoculated medium, treated with the same conditions, was also monitored. OD_600_, pH, glycerol utilization, surface tension (ST) and emulsification index (*E*_24_%) were determined with 24 h intervals. OD_600_ was assessed over time for the culture medium against a control medium. To determine the pH, glycerol utilization, ST and *E*_24_% of the culture medium, one flask was collected each day, centrifuged for 10 min at 7500 rpm and filtered through 0.22 μm membrane for biomass separation. Glycerol utilization was examined using HPLC by comparing glycerol concentration in the culture supernatant to the standard of a known concentration.^25^ Surface tension was determined at 25 °C using Krüss Force Tensiometer K6 (Krüss, Germany), calibrated with ultra-pure water according to du Nouy's ring method.^26^ To determine *E*_24_%, 2 ml of cell-free supernatant was mixed with 2 mL of *n*-hexadecane in a glass tube and vortexed for 2 min. Then, the mixture was left to stand for 24 h and the height of the liquid and emulsion layer was measured.^27^*E*_24_% was calculated according to eqn (1), as follows:1
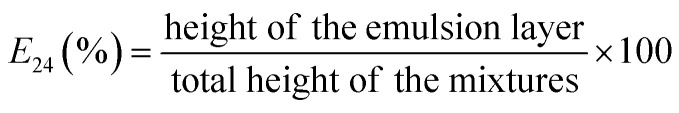


### Preparation of crude biosurfactant solution for characterization studies

To produce biosurfactant in a large-scale system composed of three 1000 mL baffled Erlenmeyer flasks containing 200 mL of MSM supplemented with 2% (w/v) of G5 glycerol, inoculum was applied in a concentration of 1% (v/v). Culture was conducted at 28 °C with rotary agitation of 180 rpm for 72 h. Then, the post-culture medium was centrifuged at 7500 rpm for 10 min and filtered through 0.22 μm membrane for biomass separation. The supernatant was lyophilized and resuspended in 20% (v/v) acetonitrile solution to obtain a biosurfactant concentration of approximately 0.17 g mL^−1^. The biosurfactant solution was purified using a solid phase extraction (SPE) system, equipped with cartridges of the Chromabond C18 SPE system (Macherey-Nagel, Germany) and eluted using an acetonitrile gradient (0%, 20%, 50%, 80% and 100% acetonitrile–water (v/v) solution). Each elution step was followed by HPLC (Shimadzu, Kyoto, Japan) performed according to the methodology described by Ciurko *et al.*^28^ to determine the composition of the solvent ensuring the most efficient biosurfactant elution. Therefore, 100 μL of the sample was dissolved in 900 μL of methanol (Chempur, Piekary Śląskie, Poland). As the mobile phase, solvents A (0.1% trifluoroacetic acid) and B (0.1% trifluoroacetic acid in acetonitrile) were applied in the following order: (% A : B v/v): 0 min (50 : 50), 5 min (20 : 80), 9 min (10 : 90), 15 min (0 : 100), 21 min (0 : 100), 24 min (50 : 50) and 25 min (50 : 50). Samples were injected in a 10 μL volume on a Hypersil GOLD column (5 μm; 4.6 × 150 mm) and eluted for 25 min. Elution was performed with a flow rate of 0.5 mL min^−1^ and detection was conducted at 210 nm wavelength. Each determination was performed in triplicate.

### Analysis of the biosurfactant chemical structure

#### Fourier-transform infrared spectroscopy (FTIR)

The structure of the purified biosurfactant was examined using FTIR and compared to the standard of surfactin (≥98.0%) (Sigma-Aldrich, Germany) and rhamnolipid (>90%) (Sigma-Aldrich, Germany). The IR spectra were observed using the IRSpirit FTIR spectrometer (Shimadzu, Kyoto, Japan) at room temperature (25 °C). The main functional groups were observed between 400 and 4000 wavenumbers (cm^−1^) at a resolution of 2 cm^−1^.

#### Electrospray ionization mass spectrometry (ESI-MS)

The biosurfactant sample was identified by electrospray ionization (ESI) mass spectrometry (MS) using a Compact Mass Spectrometer (Bruker Daltonics, Bremen, Germany) in the positive ionization mode, using the following settings: capillary voltage 3500 V, nebulizer 1.5 bar, dry gas 8 L min^−1^, dry temperature 180 °C. Data were collected for 50–3000 *m*/*z*. Next, the data obtained were processed with the Compass DataAnalysis 4.2 software package (Bruker, Bremen, Germany).

#### Determination of physicochemical properties of viscosin

According to the result of HPLC analysis, 50% (v/v) acetonitrile solution, containing the biosurfactant, was evaporated and lyophilized to remove acetonitrile impurities. Then, the fine powder of biosurfactant was examined to determine the concentration, ensuring formation of micelles. Therefore, a series of biosurfactant dilutions in a concentration range of 0–250 mg L^−1^ was analyzed in terms of ST.^26^ A KRÜSS K6 Tensiometer (KRÜSS GmbH, Germany) equipped with a 1.9 cm De Noüy platinum ring was used at room temperature (25 °C). The CMC was determined graphically by plotting the relationship between ST and biosurfactant concentration. All the measurements were made in triplicate.

In order to verify the wetting ability of the viscosin solutions, contact angle measurements were carried out using the OCA 15EC system (DataPhysics, Filderstadt, Germany), and the drop shape was analyzed with SCA20 software (DataPhysics). For each measurement, a sample droplet of 0.2 μL was placed on a solid support. Silicone, polystyrene and glass surfaces were used as supports. All measurements were made at ambient temperature (25 °C) in a saturated vapor atmosphere to reduce droplet evaporation. The contact angle value is reported as the average of ten measurements.

## Results and discussion

### Identification of bacterium

The sequence of 16S rRNA of the isolated bacterium showed 95.47% identity to *P. antarctica* CMS 35 (DSM 15318). The same score was obtained for several species within the genus *Pseudomonas*. However, results of MALDI-TOF clearly indicated species affiliation, with the identification rate of 2.13. Therefore, the isolated bacterium was affiliated to *P. antarctica*.

### The impact of post-production glycerols on *P. antarctica* 28E growth curves

Crude glycerols were identified as a proper substrate for the growth of *P. antarctica* 28E, favoring an increased growth rate. The origin of crude glycerol was as follows: G1 derived from soap production, G2–G4 derived from biodiesel production, and G5 derived from stearin production. Pure glycerol (G6) was used as a reference carbon source (Table 1).

**Fig. 1:**
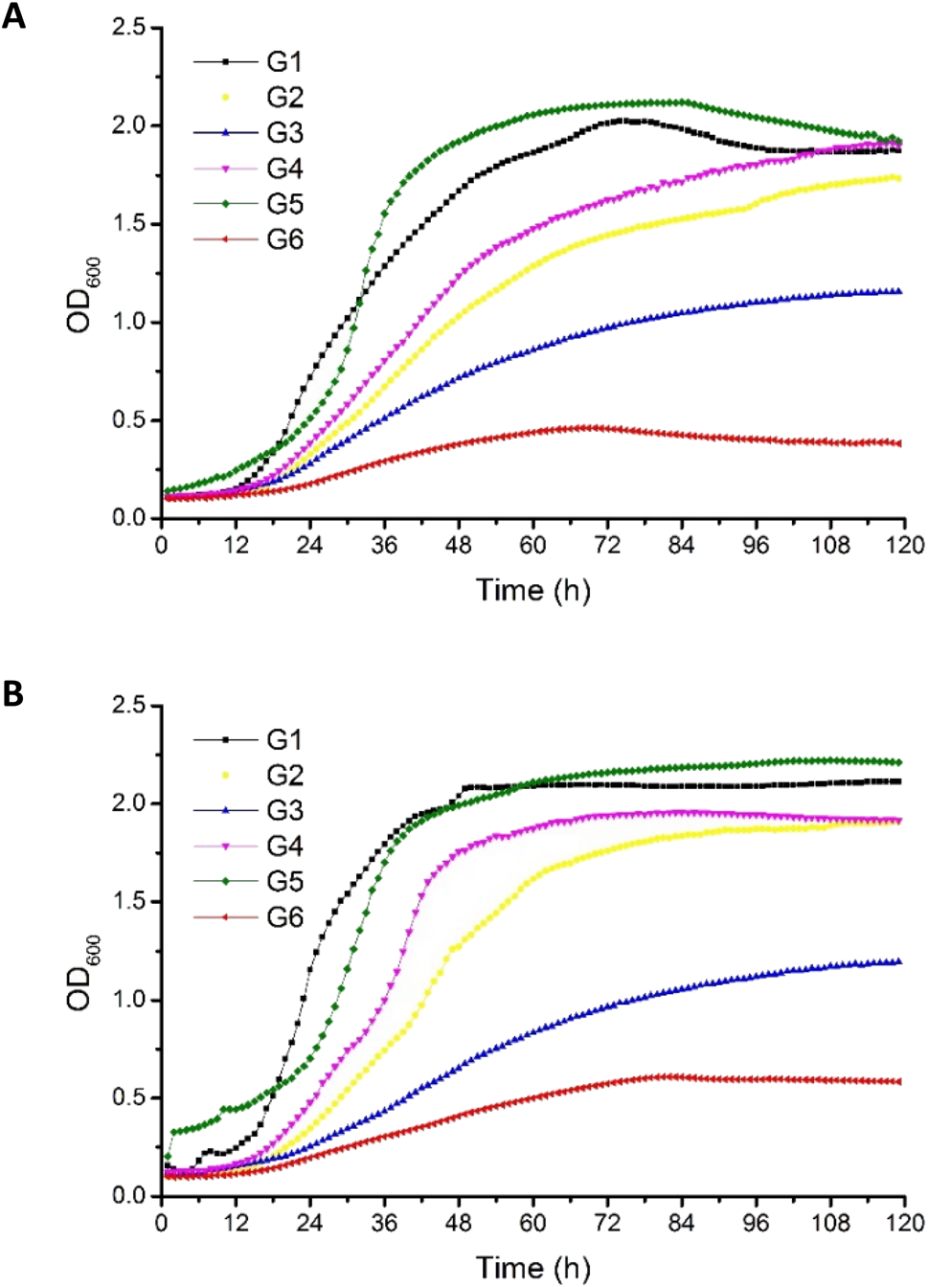
Growth of *P. antarctica* 28E on MSM medium supplemented with (A) 2% and (B) 4% crude (G1–G5) and pure glycerol (G6). Error bars represent the mean ± standard deviation of triplicate experiments.

The OD_600_ of bacterial cells in MSM medium, supplemented with G5 glycerol, was the highest among all tested variants (Fig. 1). Inversely, in the culture growing on pure glycerol, the number of bacterial cells was the most limited. This has been noted for both the 2% (w/v) (Fig. 1A) and 4% (w/v) (Fig. 1B) glycerol cultures. This should be explained by the lack of nutrients essential for bacterial growth in the reagent glycerol composition. Therefore, deficits of nitrogen and trace elements showed a negative effect for culture development. In the cultures performed with G7 glycerol addition, the *P. antarctica* 28E growth curves presented an irregular shape, suggesting the inhibitory effect of the substrate on the microbial community. We did not observe a significant increase in bacteria concentration; therefore, G7 glycerol was not included in the curves of bacterial growth (data not published) and was not considered for the future stage of studies.

The composition of G5 glycerol, recognized as the best substrate for *P. antarctica* 28E growth, was distinguished by the highest nitrogen content (Table 1). In addition, G5 glycerol was characterized by high content of iron and calcium. While the positive effect of nitrogen on microbial metabolism is well known, participating in the synthesis of amino acids, DNA, RNA and ATP among other molecules, the impact of iron and calcium is not so well explored.^29^ The high concentration of iron and calcium in G5 glycerol significantly supported the growth of *P. antarctica* 28E. Calcium, in prokaryotes, is involved in a several metabolic processes such as maintenance of cell structure, motility, cell division, gene expression and cell differentiation processes such as sporulation, heterocyst formation and fruiting body development. Therefore it is essential for bacterial colony evolution.^30^ However, the direct effect of calcium on the growth of *Pseudomonas* bacteria has not been documented so far. Therefore, this is a first report showing beneficial effect of calcium on *Pseudomonas* bacteria growth. On the other hand, several studies have highlighted the positive effect of iron, observed in our research in relation to *P. antarctica* 28E, on the growth of the genus *Pseudomonas*. Iron, applied in three different forms, *i.e.* FeSO_4_, Fe(NO_3_)_3_ and FeCl_3_, at the concentration of 0.1 mM, significantly supports the growth and biosurfactant production of *Pseudomonas citronellolis* 620C.^31^ Moreover, among the tested iron sources, the FeCl_3_ supported the microbial growth to the greatest extent. The explanation is the highest solubility of FeCl_3_ in comparison to Fe(NO_3_)_3_ and FeSO_4_. Other studies evaluated the effect of different forms and concentrations of iron on the growth rate of three biosurfactant-producing species (*P. citronellolis* (isolate 22A), *P. aeruginosa* (isolate 312A) and *P. aeruginosa* (isolate 332C)) confirmed observed in our studies promoting effect of iron on Pseudomonas growth.^32^ Several iron forms (FeCl_3_, Fe(NO_3_)_3_, Fe–EDTA, FeSO_4_, Fe^0^) at the concentration of 0.1 mM showed a positive effect on bacterial growth, increasing the cell number. The exception was iron oxide (Fe_2_O_3_), of limited solubility, which did not differ from the control.^32^ Finally, Kim *et al.*^33^ searched for a nutrient affecting the growth of *Pseudomonas syringae* pv. tomato DC3000. After a series of experiments, iron supplementation was found to induce a strong positive response for bacterial growth what is in line with our studies. Bacteria presented a dose-dependent growth enhancement. The addition of 50 μM of Fe^3+^ caused an OD_600_ change of 0.35, while supplementation with 100 μM resulted in an increase of 0.74. Based on the research described above and on the result presented in this study, the crucial role of iron on the growth of *Pseudomonas* bacteria must be emphasized. The same as the concentration applied, the bioavailability for the microbial community is of decisive importance. The post-production glycerols applied in this research, in particular G5 glycerol, meet the nutritional requirements of *P. antarctica* 28E, providing appropriate conditions for bacterial growth.

### Kinetics of biosurfactant production

**Fig. 2:**
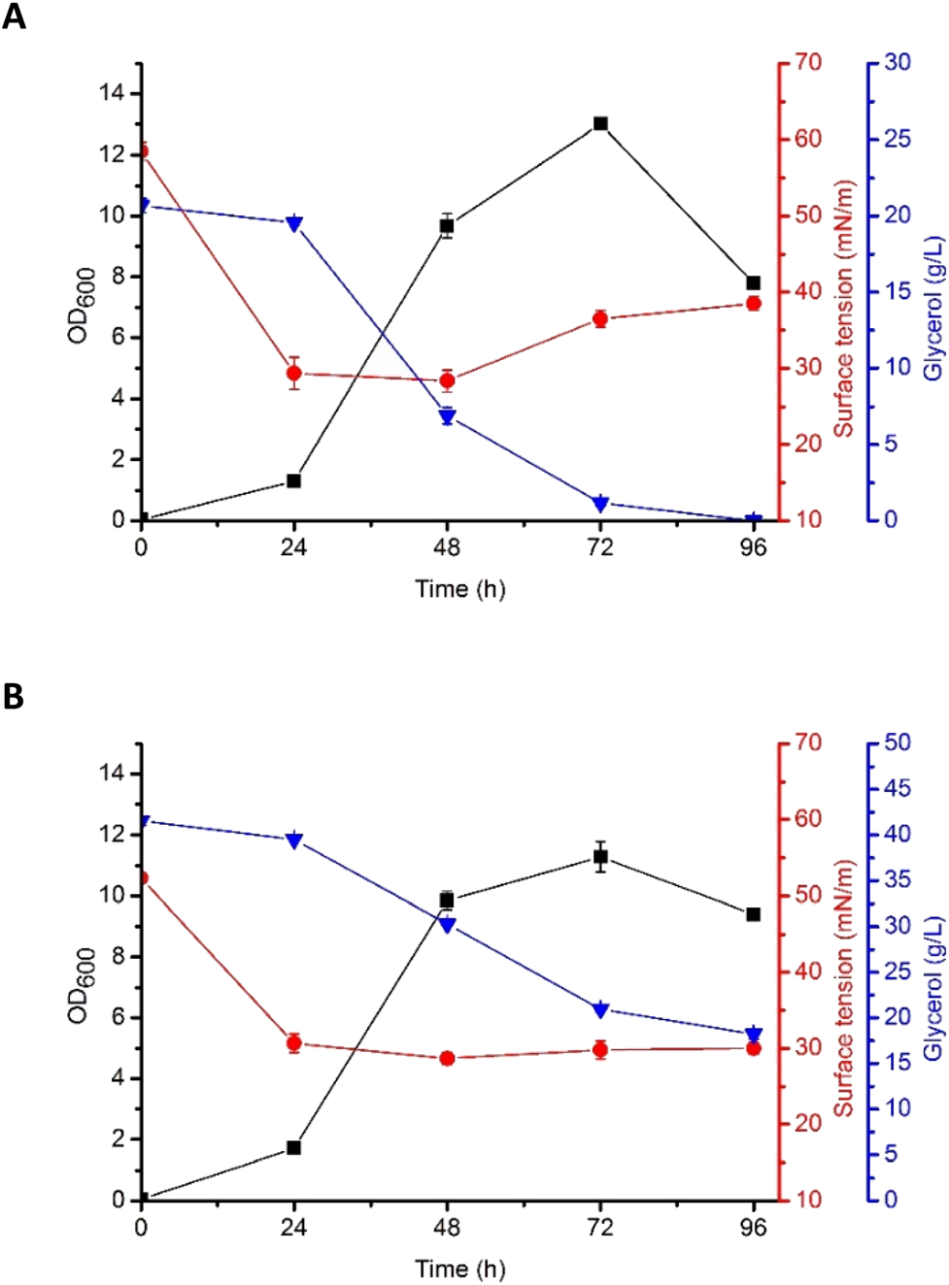
Growth curves (OD_600_), surface tension (ST, mN m^−1^), and glycerol consumption (g L^−1^) by *P. antarctica* 28E grown in MSM medium supplemented with (A) 2% and (B) 4% of crude glycerol (G5).

Based on the results described above, G5 glycerol was applied as a substrate for biosurfactant production. In the flask culture, containing either 2% (w/v) or 4% (w/v) of the substrate, *P. antarctica* 28E was growing properly, as indicated by high OD_600_ (Fig. 2). The plateau was reached after 72 h of cultivation, for both 2% (w/v) (OD_600_ 13.01 ± 0.4) and 4% (w/v) (OD_600_ 11.28 ± 1.15) glycerol concentration. Interestingly, the OD_600_ of bacterial cells was higher in 2% (w/v) glycerol medium. The explanation should be sought for in the high concentration of iron in G5 glycerol. In the 4% (w/v) glycerol medium, after 72 h of growth, the culture entered the death phase, despite the fact that almost 50% of the substrate was left (Fig. 2). In the 2% (w/v) glycerol medium the substrate was utilized within 72 h, leading to the death of the culture. According to Tsipa *et al.*^31^ the borderline between iron deficiency and toxicity is narrow. Iron is a key component of cytochromes and contributes to the Krebs cycle. In addition, it participates in the protection of the cells against superoxide radicals. However, if the iron concentration in the environment exceeds the limit, it can interact with reactive oxygen species, forming greatly damaging hydroxyl radicals. In our study, 2% (w/v) glycerol supplementation was below the nutritional requirements of *P. antarctica* 28E, while 4% (w/v) probably exceeded the limit of iron toxicity. Therefore, to ensure appropriate development of *P. antarctica* 28E culture, optimization studies should be performed. However, this is an issue for another study, while the aim of this work was to investigate the capacity of *P. antarctica* 28E to produce biosurfactant using waste substrates.

According to Moshtagh *et al.*^34^ the value of pH can affect microbial growth and reproduction significantly influencing the absorption of nutrients and activity of the enzymes engaged in several metabolic processes. In our studies, the pH of *P. antarctica* 28E culture growing in 2% (w/v) and 4% (w/v) concentration of glycerol was at a stable value of around 8 (data not shown) and it was applicable for the growth and biosurfactant synthesis of bacteria. However, numerous studies have shown that the optimal pH for biosurfactant production by *Pseudomonas* bacteria seems to be a species- or strain-dependent feature. As in our studies, the culture of *Pseudomonas aeruginosa* RS29 pH in a range of 7–8 ensured the most efficient biosurfactant production in the glycerol-based medium.^35,36^ Surprisingly, the production of biosurfactant in *Pseudomonas aeruginosa* TMN culture was the most efficient at pH 7 and a slight alkalization to pH 8 caused a dramatic change in the yield of biosurfactant production, reducing it greatly.^37^

Production of biosurfactant by *P. antarctica* 28E was indicated by the reduction of the surface tension (ST), both in 2% (w/v) and 4% (w/v) glycerol medium (Fig. 2). Significant ST reduction was observed in the early exponential phase of growth, indicating that *P. antarctica* 28E biosurfactant consists of primary metabolites, synthesized simultaneously with the formation of cellular biomass. Similar observations have been made while studying *P. aeruginosa* biosurfactant production.^38^ In *P. antarctica* 28E culture, a significant drop of the ST, from the initial value of 59 mN m^−1^ to 28 mN m^−1^ (Fig. 2A) and from 52 mN m^−1^ to 27 mN m^−1^ (Fig. 2B), respectively, in the 2% (w/v) and 4% (w/v) glycerol medium was observed. However, in the culture supplemented with 4% (w/v) glycerol, ST dropped significantly during 24 h and reached a stable value of approximately 27 mN m^−1^, while in the 2% (w/v) glycerol medium, after ST decline, a slight increase was observed. It indicated reduction of the biosurfactant concentration in the 2% (w/v) glycerol medium. The ST increase occurred after glycerol stores were depleted. Therefore, to provide the bacteria with nutrients, the already produced biosurfactants could possibly be hydrolyzed.

A variety of microorganisms of the genus *Pseudomonas* produce biosurfactants that are diverse in chemical composition. The nature and amount of the biosurfactant produced depends on the species of *Pseudomonas* and the various nutritional factors available for their growth (Table 2).

**Table 2:** Potential biosurfactants with their producing bacterial strains

Biosurfactant type	Strain	By-products/carbon sources	Maximum yield of production (g L^−1^)	Reference
**Glycolipids**				
Rhamnolipids	*Pseudomonas aeruginosa* E03-40	Soybean oil/glycerol	42.1	39
Rhamnolipids	*Pseudomonas aeruginosa* J4	Glucose/kerosene/glycerol/olive oil/sunflower oil/grape seed oil	3.6	40
Rhamnolipids	*Pseudomonas aeruginosa* EM1	Glucose/glycerol/sucrose/hexane/olive oil/oleic acid/soybean oil	12.3	41
Rhamnolipids	*Pseudomonas aeruginosa* RS6	Waste glycerol	2.73	42

**Lipopeptides**				
Pseudofactin	*Pseudomonas fluorescens* BD5	Glucose/glycerol	1.19/0.01	43 and 44
Putisolvin	*Pseudomonas putida* PCL1445	Glycerol	—	45
Amphisin	*Pseudomonas* sp. DSS73	Glucose	—	46
Syringomycin	*Pseudomonas syringae* B3A-R	Glucose	—	47
Viscosin	*Pseudomonas libanensis* M9-3	Glucose	0.15	48

Glycerol is a frequently chosen substrate in the research on biosurfactant production in the *Pseudomonas* genus, while most of these studies concern rhamnolipid synthesis.^35,36^ Biodiesel side stream waste glycerol was applied for biosurfactant production using *Pseudomonas aeruginosa* RS6. At optimal fermentation conditions, ST declined from 72.13 mN m^−1^ to 29.4–30.4 mN m^−1^; therefore biosurfactant activity was reported to be better than that of some chemical-based surfactants and similar to the activity observed in our studies.^49^ Corresponding to our research ST drop was observed in another study, where glycerol was applied as a substrate for anaerobic production of rhamnolipids. Three strains of *P. aeruginosa* (SG, L6-1 and FA1) reduced the ST at different substrate concentrations from 63 mN m^−1^ to about 32 mN m^−1^. In addition to glycerol, its intermediate, *i.e.* 1,2-propylene glycol, as well as crude glycerol supported *P. aeruginosa* strains to anaerobically produce rhamnolipids, reducing the surface tension of the culture to about 31 mN m^−1^ and below 35 mN m^−1^ respectively.^50^ Finally, in the research performed by Silva *et al.*^51^ rhamnolipid production in the 3% glycerol culture of *P. aeruginosa* UCP0992 resulted in an ST reduction to the value of 27.4 mN m^−1^ as it was observed in our study. Dubern and Bloemberg^52^ detected a similar ST drop, while describing production of putisolvin, the lipopeptide biosurfactant of *Pseudomonas putida* strain PCL1445. In the medium supplemented with 2% glycerol, ST decreased from 54 mN m^−1^ initially to 29 mN m^−1^, what is in line with our studies. Reduction of the ST observed in our research as well as described literature examples showed post-production glycerol as a suitable substrate for the production of *Pseudomonas* biosurfactant.

Biosurfactant production in the *P. antarctica* 28E culture affected emulsifying properties of the cultivation medium was indicated in addition by the growing *E*_24_%. In the medium supplemented with 2% as well as 4% glycerol, emulsification index (*E*_24_%) reached a maximum value of 33% (data not shown). However, obtained result was lower than that observed in the research of da Rosa *et al.*^53^ where in the optimal conditions for rhamnolipid production *E*_24_% reached the value of 61%. In another study, where the halophilic bacterium *Pseudomonas stutzeri* BK-AB12 was cultivated in medium containing 3% glycerol, an increase of *E*_24_% reach the higher value of 53.33%. Finally, the cell-free supernatant of *P. aeruginosa* JBK1 culture, cultivated in 3% biodiesel derived waste glycerol medium, exhibited stable emulsifying properties with all the hydrocarbons tested. The best *E*_24_% results; 62% obtained in relation to kerosene and xylene and 60% obtained using hexadecane^38^ were almost twice as high as in our research. Against the background of the presented results, the *E*_24_% of *P. antarctica* 28E culture supernatant is relatively low. According to Jain *et al.*^54^ high molecular weight biosurfactant acts as an emulsion stabilizer, while low molecular mass compounds are unable to create stable emulsions. This indicates the presence of the low molecular weight biosurfactant in *P. antarctica* 28E culture medium.

### Report on biosurfactant characterization studies

#### Lipopeptide nature of *P. antarctica* 28E biosurfactant indicated by a Fourier-transform infrared spectroscopy (FTIR)

**Fig. 3:**
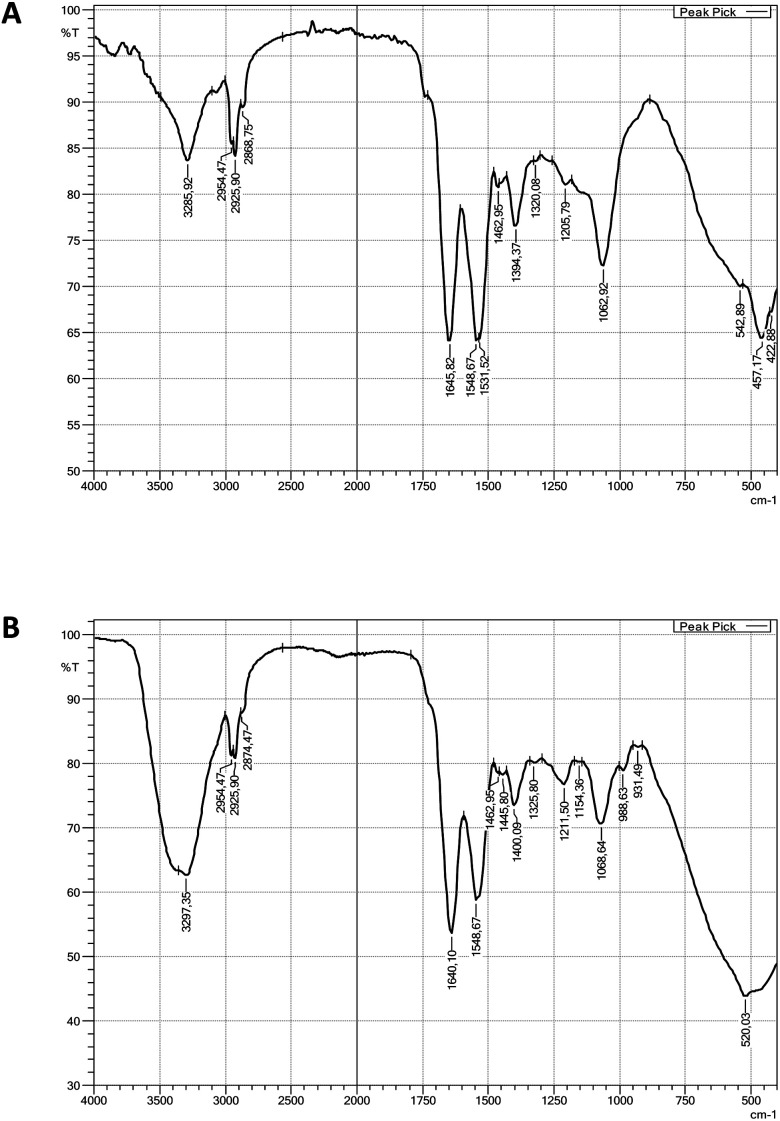
Fourier transform infrared spectroscopy (FTIR) spectra: (A) viscosin, (B) surfactin standard.

FTIR was used in order to verify the chemical nature of *P. antarctica* 28E biosurfactant. We observed the characteristic peaks at 3286 cm^−1^ and 1646 cm^−1^ corresponding to the N–H and CO–N stretching vibrations respectively (Fig. 3A). In addition, absorption bands at 2954 cm^−1^, 2926 cm^−1^ and 2869 cm^−1^ assigned to asymmetric C–H stretching vibrations were detected. The presence of aliphatic chains (–CH_3_, –CH_2_–) was confirmed by peaks at 1463 cm^−1^ and 1394 cm^−1^. Another peak, detected at 1063 cm^−1^ indicated the presence of C–O bonds in the biosurfactant structure. Summing up, the observed peaks displayed the presence of aliphatic hydrocarbons attached to a peptide moiety. Therefore the biosurfactant of *P. antarctica* 28E was classified as a lipopeptide. Corresponding peaks were observed on the surfactin standard spectrum (Fig. 3B). Moreover, Liu *et al.*,^55^ Thavasi *et al.*^56^ and Pardhi *et al.*^57^ detected similar absorption peaks while studying lipopeptides secreted by *P. aeruginosa* SNP0614, *P. aeruginosa* and *Pseudomonas guguanensis* D30, respectively.

#### Structural characterization of *P. antarctica* 28E lipopeptide

**Fig. 4:**
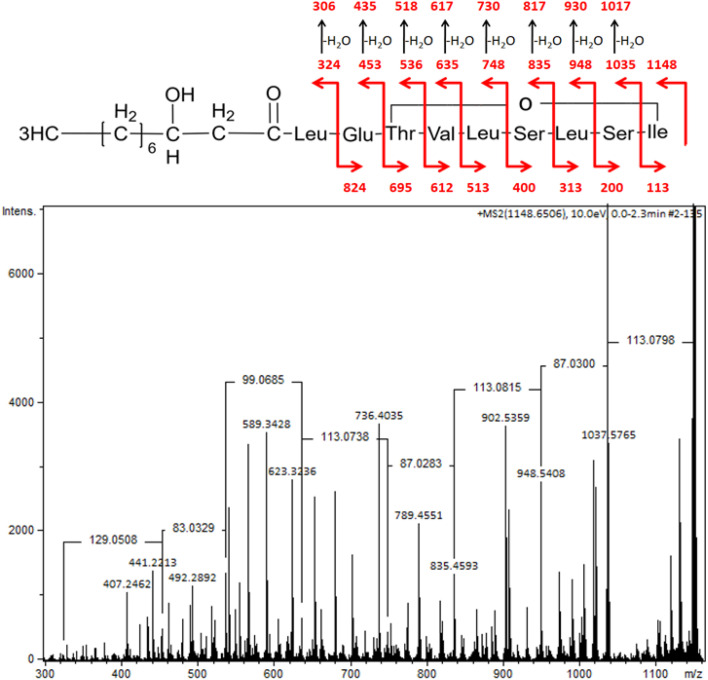
MS/MS spectra of pseudomolecular ion of viscosin at *m*/*z* 1148 (M + [Na^+^]) with the structure fragmentation.

In order to identify the lipopeptide of *P. antarctica* 28E, ESI-MS was conducted. The pseudomolecular ions at *m*/*z* 1148.65 (M + [Na^+^]) and 1124.69 (M − [H^+^]), obtained respectively using positive and negative ionization mode, allowed the lipopeptide to be identified as viscosin. The MS/MS spectra of the parent ion at *m*/*z* 1148.65 (M + [Na^+^]) displayed the set of daughter ions, formed as a result of peptide bonds' fragmentation, as presented in Fig. 4.

A similar spectrum of viscosin fragmentation was observed in the work of Laycock *et al.*,^58^ where protonated fragmentation ions at *m*/*z* 595, 496, 413 and 284 were detected. In our work, corresponding ions, which were sodium adducts (M + [Na^+^]) of *m*/*z* at 617, 518, 435 and 306, were noted. The MS/MS data presented in the work of Rokni-Zadeh *et al.*,^59^ where structural studies on the white line-inducing principle (WLIP) were performed, are in line with our results. WLIP is a cyclic lipopeptide with a molecular weight of 1125 Da composed of an N-terminal β-hydroxydecanoic acid and a peptide moiety of nine amino acids. It is structurally identical to viscosin except for the chirality of leucine in position 5 that it is d- in WLIP and l- in viscosin.^60^ MS/MS spectra of WLIP displayed signals at *m*/*z* 995.6, 908.6, 795.5, 595.4, 496.3, 413.3 and 284.2 (M + [H^+^]), corresponding to the fragmentation ions obtained in our studies. Finally, the MS/MS spectrum obtained in our research corresponded to the one published while studying antifungal activity of viscosin, secreted by frog skin bacteria.^61^ Therefore, lipopeptide biosurfactant obtained in our studies were identified as viscosin.

**Fig. 5:**
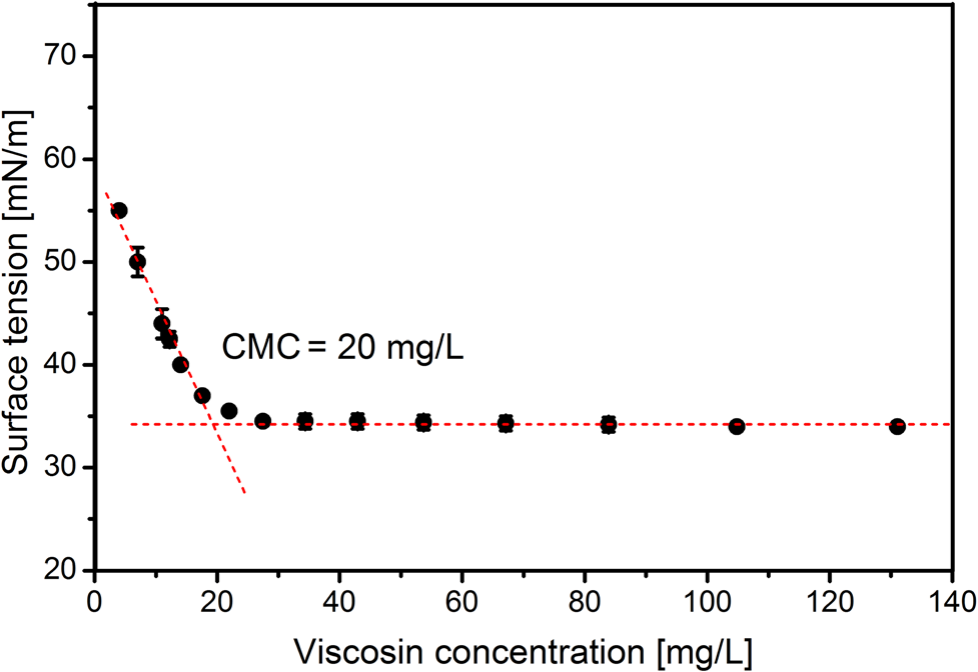
Critical micelle concentration (CMC) of purified viscosin.

As the final stage of this study the CMC of viscosin was determined. The CMC reached 20 mg L^−1^ (Fig. 5), which is in line with other results, *i.e.* 15 mg L^−1^, 50 mg L^−1^ and 54 mg L^−1^, presented in the work of Hamley,^62^ Portet-Koltalo *et al.*^63^ and Saini *et al.*,^21^ respectively.

The viscosin biosurfactant was studied for any effect on changes in the contact angle on hydrophobic and hydrophilic surfaces. Silicone and polystyrene surfaces, which have high hydrophobicity, are used regularly in biomedical industries. The largest reduction in contact angle was observed on silicone (from 92° to 77°) and polystyrene (from 75° to 61°) at 0.02% (w/v) viscosin (Table 3).

**Table 3:** Contact angles (degrees) for viscosin obtained from *P. antarctica* 28E on different surfaces, glass, polystyrene and silicone

Sample	Contact angle (°)							
	Viscosin concentration (mg L^−1^)							
	0	3	6	10	20	60	100	200
Glass	6 ± 1	6 ± 0	6 ± 1	6 ± 0	5 ± 0	5 ± 1	6 ± 0	5 ± 1
Polystyrene	75 ± 1	73 ± 2	73 ± 1	70 ± 1	67 ± 2	66 ± 2	61 ± 2	61 ± 2
Silicone	92 ± 2	89 ± 1	85 ± 2	82 ± 2	80 ± 1	80 ± 2	78 ± 2	77 ± 2

However, on a highly hydrophilic surface like glass, there was no significant reduction in contact angle values (from 6° to 5°) for 0.02% (w/v) viscosin solution (Table 3). For comparison, Al-Wahaibi *et al.*^64^ reported changes in the wettability of a hydrophobic surface from 59° to 29° by biosurfactant produced by *B. subtilis* B30. Al-Sulaimani *et al.*^65^ also reported that biosurfactant produced by *B. subtilis* W19 changed the contact angle of distilled water from 70.6° to 25.3° at 0.25% (w/v) biosurfactant. In conclusion, our results suggest that the viscosin changed the wettability of hydrophobic surface toward more water-wet, which is beneficial during enhanced oil recovery (EOR) applications.

## Conclusion

Selection of suitable substrates for lipopeptide production by *Pseudomonas* species is of great importance. The proper substrate should be inexpensive and renewable, and provide adequate conditions for bacterial growth. Our work has shown that glycerol, as the sole carbon and energy source, has great potential for increasing cell growth. Significant impacts of iron, calcium and nitrogen ions on *P. antarctica* 28E culture growth and biosurfactant production were observed. Importantly, iron concentration was found to stimulate or inhibit *Pseudomonas* growth, suggesting the importance of its adjustment in the medium. The pH value was also found to significantly affect the biosurfactant production. Furthermore, *P. antarctica* 28E is able to produce a lipopeptide viscosin (1125 Da). Moreover, pH 8 is suitable for lipopeptide production. Viscosin demonstrated significant activity, reducing ST considerably in 2% (w/v) and 4% (w/v) glycerol medium. In addition, high viscosin activity was manifested by the low CMC, determined to be 20 mg L^−1^, which is in line with previously published data. The low CMC as well as the production using cheap, waste substrates supports the economic advantages for possible future large-scale viscosin application.

This study provides the first evidence that viscosin can be produced using *P. antarctica* 28E in a culture medium containing crude glycerol. To reduce the costs of biosurfactant production, stearin-derived glycerol was used as a promising cheap and renewable carbon source. The properties of the biosurfactant obtained have potential application as a cleaning and emulsifying agent in the pharmaceutical and food industry and/or bioremediation of hydrocarbon-contaminated sites.

## Author contributions

DC: conceptualization, investigation, formal analysis, validation, visualization, methodology, writing—original draft. AC, MK, HC-I and IP: investigation, methodology. AKU: investigation. AF: writing—review & editing. TJ: conceptualization, formal analysis, validation, resources, supervision, investigation, writing—review & editing. All authors have read and agreed to the published version of the manuscript.

## Conflicts of interest

The authors declare that they have no known competing financial interests or personal relationships that could have appeared to influence the work reported in this paper.

## Supplementary Material
